# Hydatid Cyst Protoscolices Induce Cell Death in WEHI-164 Fibrosarcoma Cells and Inhibit the Proliferation of Baby Hamster Kidney Fibroblasts *In Vitro*

**DOI:** 10.1155/2012/304183

**Published:** 2012-01-31

**Authors:** Hossein Yousofi Darani, Narges Soozangar, Soliman Khorami, Fatomeh Taji, Mortaza Yousofi, Hedayatollah Shirzad

**Affiliations:** ^1^Department of Parasitology, Cell and Molecular Research Center, Faculty of Medicine, Shahrekord University of Medical Sciences, Shahrekord, Iran; ^2^Department of Parasitology & Mycology, Faculty of Medicine, Shahrekord University of Medical Sciences, Shahrekord, Iran; ^3^Student Research Committee, Shahrekord University of Medical Sciences, Shahrekord, Iran; ^4^Medical Plants Research Center, Shahrekord University of Medical Sciences, Shahrekord, Iran; ^5^Department of Immunology, Cell and Molecular Research Center, Shahrekord University of Medical Sciences, Shahrekord, Iran

## Abstract

Both *in vitro* and *in vivo* models have demonstrated that some parasites can interfere with tumor cell growth. The present study investigates the anticancer activity of hydatid cyst protoscolices on WEHI-164 fibrosarcoma cells and baby hamster kidney (BHK) fibroblast cells *in vitro*. Those above two cell types were treated with live hydatid cyst protoscolices or left untreated for control groups. After 48 h, lactate dehydrogenase (LDH) and cell counts were assayed for both treated cells and control groups. Following treatment with hydatid cyst protoscolices, cell proliferation of both cell types was inhibited, and lysis of fibrosarcoma cells increased. Based on these results, it appears that hydatid cyst protoscolices have strong anticancer activity, and additional studies are needed to further clarify the mechanisms of this activity.

## 1. Introduction

A hydatid cyst contains the larval stage of the tapeworm, *Echinococcus granulosus*, which is a parasite responsible for the zoonotic illness, echinococcosis. This disease is also known as hydatid disease or hydatidosis in human and livestock. It has previously been demonstrated that some parasitic infections induce antitumor activity against certain types of cancers [1–6]. In addition, mice immunized with *Toxoplasma gondii* tachyzoites, or *Toxocara canis* egg antigens, that were subsequently challenged with fibrosarcoma cells, showed a reduction in solid tumor growth compared with mice that were not immunized, yet were challenged with the same cells [7]. An inhibition of tumor cell proliferation has also been associated with certain types of parasites [8]. These findings support the hygiene theory whereby a decrease in exposure to infectious agents early in life is predicted to increase susceptibility to allergies, and perhaps autoimmune diseases [9–11]. Protoscolices are small masses with four suckers and a double row of hooks and each is capable of developing into an adult worm in the intestine of the final host. Akgül et al. showed that in a large retrospective study of patients with hydatid disease, the prevalence of cancer was significantly lower than in normal subjects [12]. In another study, it has been shown that cancer-associated mucin-type O-glycosylated antigens such as Tn and sialy-Tn antigen were presented in hydatid cyst protoscolices and adult stage of *Echinococcus granulosus *[13]. These common antigens may have a role in induction of immunological cross-reactions between cancers and hydatid cyst. To explore the anticancer activity of this parasite, the effects of hydatid cyst protoscolices on the proliferation and death of mouse fibrosarcoma cells, and baby hamster kidney (BHK) fibroblast cells, was investigated.

## 2. Materials and Methods

### 2.1. Parasite Used

In this experimental study, *Echinococcus granulosus* hydatid cysts were collected from sheep or cattle from a slaughter house in Isfahan, Iran. Cysts were examined, and any protoscolices present were aspirated and collected. Protoscolices were then centrifuged at 2000 ×g, for 2 min, and the supernatant was discarded. 10 mL saline was added to the remaining protoscolices in a test tube and centrifuged as above, and the supernatant was discarded. One more wash with saline was given to the protoscolices and then used immediately.

### 2.2. Culturing of Tumor Cells

Two cell lines including BHK fibroblast cells, as well as WEHI-164 and Balb/C mouse fibrosarcoma cells, were provided by the Pasture Institute (Tehran, Iran). Cells were cultured in Dulbecco's modified Eagle's medium (DMEM; Sigma) supplemented with 20 mM HEPES, 0.2 mM L-glutamine, 50 *μ*M 2-mercaptoethanol, 0.15% sodium bicarbonate, 50 *μ*g/mL gentamicin, and 10% fetal calf serum (FCS), as reported previously [14]. Tumor cell viability was assayed using trypan blue staining.

### 2.3. Lactate Dehydrogenase (LDH) Assay

The *in vitro* lysis of WEHI-164 tumor cells by parasites was assayed by detecting the release of LDH as reported previously [14]. Briefly, 1 × 10^5^ WEHI-164 cells were incubated with hydatid cyst protoscolices at 37°C in 5% CO_2_ and 90% relative humidity. At the indicated time points, cells were centrifuged, and 50 *μ*L of supernatant was harvested. The amount of LDH present in each sample was estimated using the kit (Parsazmoon,Tehran, Iran) according to the manufacturer's instructions. Controls included untreated WEHI-164 cells.

### 2.4. Experimental Design

For each experiment, six culture flasks containing 10 mL culture medium with 1 × 10^5^ freshly prepared and viable WEHI-164 or BHK cells were prepared. Flasks A, B, and C were treated with 10, 50, or 100 live hydatid cyst protoscolices, respectively, while the remaining three flasks were left untreated as control samples. Cell count and LDH concentrations were determined for each flask after 48 h. Each experiment was performed in triplicate.

## 3. Results

**Figure 1:**
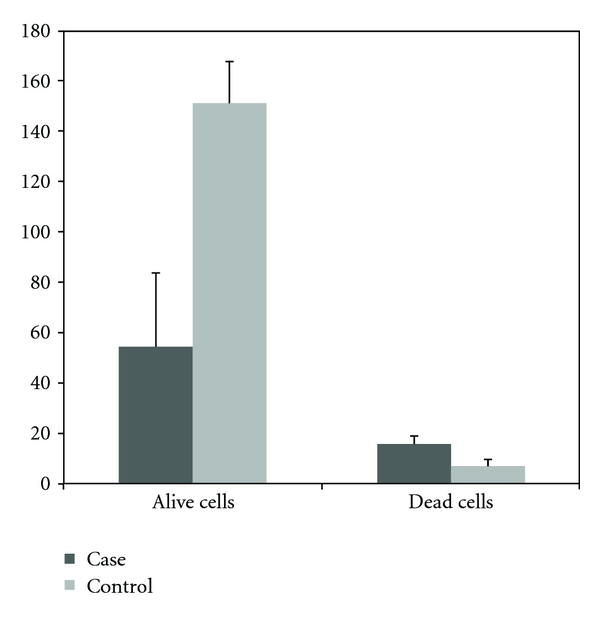
The the number of viable or dead cells (×10^5^) in flasks of WEHI-164 fibrosarcoma cells 48 h after treatment with 100 hydatid cyst protoscolices compared to control cells (mean of triplicate performance).

**Figure 2:**
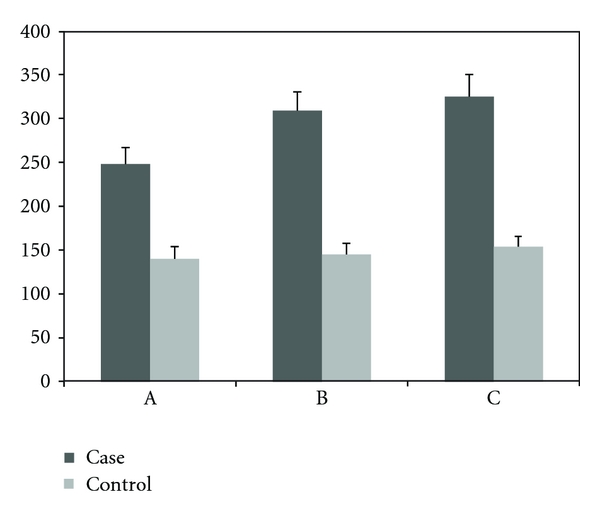
An estimate of LDH (units/L) in flasks of WEHI-164 fibrosarcoma cells 48 h after treatment with 10 (A), 50 (B), or 100 (C) hydatid cyst protoscolices compared with control cells (mean of triplicate performance).

The proliferation and viability of WEHI-164 fibrosarcoma cells treated with *Echinococcus granulosus* hydatid cyst protoscolices (Figure 1) were compared with untreated WEHI-164 fibrosarcoma cells (control group). In these assays, when 10 protoscolices were incubated with WEHI-164 cells, no obvious effect was observed. However, when 50 or 100 protoscolices were added, cell proliferation was inhibited and cell lysis increased. The cell counts for these assays are presented in Table 1 and Figure 1, while the LDH assay results are presented in Figure 2.

**Figure 3:**
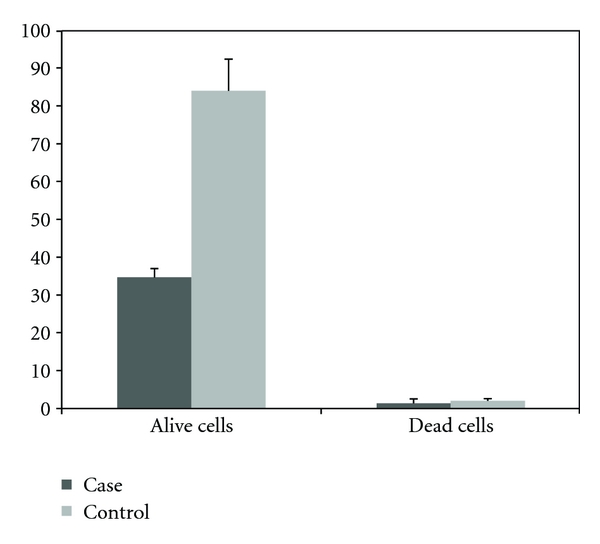
The number of viable or dead cells (×10^5^) in flasks of BHK fibroblast cell 48 h after treatment with 100 hydatid cyst protoscolices compared to control cells (mean of triplicate performance).

In similar experiments, the effects of hydatid cyst protoscolices on the proliferation and viability of BHK fibroblast cells were evaluated in comparison with untreated BHK cells (control group). A significant difference between the number of viable cells detected in treated and control groups was observed. However, the difference in the number of dead cells associated with each group was not significant (Table 2 and Figure 3).

## 4. Discussion

The results of this study indicate that hydatid cyst protoscolices inhibit the proliferation of WEHI-164 and BHK cells and have the capacity to induce cell death in WEHI-164 cells *in vitro*. These results are consistent with previous studies that have shown some parasitic and microbial infections interfere with tumor growth to mediate anticancer activities [1–8]. For example, different strains of *Trypanosoma cruzi* produce a reagent that selectively damages human cancer cells *in vitro* and reduces tumor growth *in vivo* [2, 4, 15]. Therefore, the anticancer activity observed for hydatid cyst protoscolices is consistent with these previous studies. In Turkey, 1/1200 patients with differing hematologic neoplastic diseases have experienced acute leukemia and liver hydatidosis concomitantly [16]. Moreover, given that hydatid cysts are endemic in Turkey, this rate of incidence is also consistent with the findings of the present study. In another investigation, Akgül et al. showed that in a large retrospective study of patients with hydatid disease, the prevalence of cancer was significantly lower than in normal subjects [12].

The anticancer activity of hydatid cyst protoscolices may be due to surface antigen activity, or the excretory-secretory products of the parasite. In work by Alvarez Errico et al., the carcinoma-associated Tn antigens were detected in *Echinococcus granulosus* protoscolices and circulating Tn antigens in hydatid patients [13]. These antigens have been implicated in metastasis of tumor cells, and a direct link has been shown between carcinoma aggressiveness and the density of them [17]. So these antigens may be involved in the inhibition of WEHI-164 or BHK cells.

However, the mechanisms involved in the inhibition of cell proliferation and induction of cell lysis mediated by hydatid cyst protoscolices are not clear and require further investigation.

## Figures and Tables

**Table 1:** The number of dead or alive cells in flasks with WEHI-164 fibrosarcoma cells following 48 hours of treatment with 10 (A), 50 (B), or 100 (C) hydatid cyst protoscolices (case groups) or left intact (control group) (mean of triplicate performance).

	Case groups mean			Control group	
	A (mean)	B (mean)	C (mean)	Controls mean	*P*
Number of alive cells (×10000)	121 ± 2	106.5 ± 7.8	54.3 ± 29.5	151 ± 16.5	0.003
Number of dead cells (×10000)	10.3 ± 2.9	12.5 ± 2.9	15.8 ± 3.1	7 ± 2.6	0.024

**Table 2:** The number of dead or alive cells in flasks with BHK cells following 48 hours of treatment with 10 (A), 50 (B), or 100 (C) hydatid cyst protoscolices (case groups) or left intact (control group) (mean triplicate performance).

	Case groups mean			Control group	
	A (mean)	B (mean)	C (mean)	Controls mean	*P*
Number of alive cells (×10000)	64 ± 10.58	50 ± 9.16	34.66 ± 2.30	84 ± 8.3	0.018
Number of dead cells (×10000)	2 ± 1	1.33 ± 1.15	1.33 ± 1.15	2 ± 0.5	0.532
